# Histone code, human growth and cancer

**DOI:** 10.18632/oncotarget.435

**Published:** 2012-01-25

**Authors:** Francesco Crea

**Affiliations:** University of Pisa, Division of Pharmacology Department of Internal Medicine

Epigenetics refers to all heritable changes, which are not dependent on alterations of DNA primary structure [1]. Among them, histone post-translational modifications (HPTMs) play a crucial role in regulating gene expression. Histones are core chromatin components, organized in cylindrical structures. The nucleosome is the fundamental chromatin unit: it is made of appreciatively 150 bp. DNA wrapped around a cylindrical histone core [2]. Histone N-terminal tails protrude from this compact structure, and may be modified by several HPTMs (acetylation, methylation, phosphorylation…). Each modification occurs on a specific residue, and is mediated by an enzymatic complex. Since HPTMs dictate DNA-chromatin binding and gene activity, it has been proposed that a complex histone code orchestrates gene expression in mammalian cells [2]. HPTMs have been shown to control several developmental processes, and are deregulated in many human diseases, including cancer. Among HPTMs, methylation is particularly interesting, because it can activate or silence gene expression, depending on target amino acidic residue. For example, histone H3-Lys27 trimethylation, mediated by Polycomb member EZH2, is known to silence gene expression; while histone H3-Lys36 dimethylation, mediated by NSD1, is an activating mark [3]. HPTMs appear to be crucial for stem cell self-renewal and tissue specification, and may be involved in some developmental diseases [2].

Inactivating NSD1 gerrmline mutations have been shown to occur in individuals affected by Sotos syndrome (SS), characterized by accelerated pre- and post-natal growth, macrocephaly, advanced bone age and developmental delay [4]. Interestingly, NSD1 mutations are rare in similar but less frequent overgrowth conditions, including Weaver syndrome (WS). WS shares with SS the above mentioned hallmarks. However, individuals with SS are less likely to show increased birth weight than patients affected by WS, and in childhood tend to be taller and thinner [5]. Moreover, facial appearance is different in these two syndromes. Two recent papers [6, 7] found a possible genetic explanation for these slight and apparently questionable clinical differences. Both studies started from a whole-genome screening of possible mutations in a small number of subjects with WS. Surprisingly, both studies found that EZH2 gene was frequently mutated in those patients. The study published in the December issue of *Oncotarget* also confirmed the presence of EZH2 mutations in a larger set of patients affected by overgrowth syndrome [7]. Most mutations appear to occur *de novo* and are predicted to inactivate the gene, through deletion or alteration of the catalytic domain. Thus, EZH2 alterations may somehow cause WS. Due to the role of EZH2 in maintaining stem cell self-renewal and delaying tissue specification [2], it is conceivable that inactivation of this gene triggers the accelerated bone maturation observed in WS. Intriguingly, EZH2 is also involved in brain stem cell differentiation [2], and most WS patients display learning disabilities. Thus, the putative relationship between histone methylation and brain development may open the way to a new perspective for epigenetic research.

It is less clear whether EZH2 mutations predispose for neoplasia. Indeed, 2 of 19 patients with overgrowth syndrome and EZH2 mutation developed a tumor. One of them developed neuroblastoma and lymphoma. Both neoplasms have been previously associated with EZH2 *hyperactivation* [8, 9]. Interestingly, this patient displayed a missense mutation in the catalytic domain, which may not simply inactivate protein function. For example, a coordinated activity of wild-type and mutant EZH2 was shown to drive histone H3-Lys27 trimethylation in human B-cell Lymphomas [9]. Another obscure issue is why mutations in two counteracting epigenetic effectors (NDS1 and EZH2) cause very similar syndromes.

Future studies should shed new light on the complex relationship between HPTMs and human development. In particular, the molecular function of EZH2 and NSD1 in bone development should be addressed. Moreover, histone methylation and demethylation may be modulated by novel small molecule inhibitors [10, 11], which may pave the way to a therapeutic approach for those rare syndromes.
